# Supplementary material for: Anticipatory Postural Adjustments and kinematic arm features when postural stability is manipulated
Source: PeerJ. 2018 Mar 15;6:e4309. doi: 10.7717/peerj.4309 (PMC5857349; doi:10.7717/peerj.4309)
Supplement: Table S1 — (APA, anticipatory postural adjustments; RT, reaction time; VP, arm velocity profile). Arbitrary values given in each vertical column indicate the presence (1) or absence (0) of APA. For the RT, values are presented as percentage of a maximum arbitrary value and for VP, the value 0.5 represents the complete symmetry on acceleration and deceleration time. [file peerj-06-4309-s001.docx]

The supplementary table sums up our predictions about the interaction between equilibrium constraints, APAs and kinematic features of the upper-limb.

Supplementary table. Predictive values of Kinematic parameters varying across postural stability. (APA, anticipatory postural adjustments; RT, reaction time; VP, arm velocity profile). Arbitrary values given in each vertical column indicate the presence (1) or absence (0) of APA. For the RT, values are presented as percentage of a maximum arbitrary value and for VP, the value 0.5 represents the complete symmetry on acceleration and deceleration time).

|  | APAs stabilizer | APA accelerators | RT | VP |
| --- | --- | --- | --- | --- |
| Stable Posture | **0** (APAs not required) | **1** (forward CoM acceleration facilitates target reaching ) | **50%** (easy equilibrium control due to stable posture induces simple motor plan) | **0.5** (easy control does not require longer duration of arm deceleration) |
| Unstable Posture | **1** (APAs required) | **1** (forward CoM acceleration facilitates target reaching ) | **100%** (challenging equilibrium control induces complex planning) | **0.4** (challenging control requires longer Deceleration duration) |
